# Comparative Analysis of Composition, Texture, and Sensory Attributes of Commercial Forms of Plant-Based Cheese Analogue Products Available on the Irish Market

**DOI:** 10.3390/foods14152701

**Published:** 2025-07-31

**Authors:** Farhan Ali, James A. O’Mahony, Maurice G. O’Sullivan, Joseph P. Kerry

**Affiliations:** School of Food and Nutritional Sciences, University College Cork, T12 TP07 Cork, Ireland; 122110944@umail.ucc.ie (F.A.); sa.omahony@ucc.ie (J.A.O.); maurice.osullivan@ucc.ie (M.G.O.)

**Keywords:** plant-based cheese, dairy cheese, microstructure, meltability, rheology, texture, sensory evaluation

## Abstract

The increasing demand for plant-based foods has led to significant growth in the availability, at a retail level, of plant-based cheese analogue products. This study presents the first comprehensive benchmarking of commercially available plant-based cheese analogue (PBCA) products in the Irish market, comparing them against conventional cheddar and processed dairy cheeses. A total of 16 cheese products were selected from Irish retail outlets, comprising five block-style plant-based analogues, seven slice-style analogues, two cheddar samples, and two processed cheese samples. Results showed that plant-based cheese analogues had significantly lower protein content (0.1–1.7 g/100 g) than cheddar (25 g/100 g) and processed cheese (12.9–18.2 g/100 g) and lacked a continuous protein matrix, being instead stabilized largely by solid fats, starch, and hydrocolloids. While cheddar showed the highest hardness, some plant-based cheeses achieved comparable hardness using texturizing agents but still demonstrated lower tan δ_max_ values, indicating inferior melting behaviour. Thermograms of differential scanning calorimetry presented a consistent single peak at ~20 °C across most vegan-based variants, unlike the dual-phase melting transitions observed in dairy cheeses. Sensory analysis further highlighted strong negative associations between PBCAs and consumer-relevant attributes such as flavour, texture, and overall acceptability. By integrating structural, functional, and sensory findings, this study identifies key formulation and performance deficits across cheese formats and provides direction for targeted improvements in next-generation PBCA product development.

## 1. Introduction

Environmental and health considerations are contributing strongly to increasing demand for plant-based food offerings in the diet [1,2]. The global market for plant-based dairy products is anticipated to expand from USD 30.49 billion in 2024 to USD 62.03 billion by 2031, with a compound annual growth rate (CAGR) of 10.7% [3]. Specifically, the plant-based cheese market, which was valued at USD 1.4 billion in 2023, is projected to grow at a CAGR of over 16.0% by 2030 [4]. This significant growth highlights the rising consumer demand for plant-based offerings, diversity, and choice in their purchasing choices.

Cheese, a fundamental component of diets worldwide, is celebrated for its wide range of flavours, textures, and versatility. Among the most popular types is cheddar, which is traditionally made from pasteurized cow’s milk, using calf rennet or its alternatives for coagulation [5]. Processed cheese, on the other hand, is created by melting and heating natural cheese blends with the addition of emulsifying salts and other ingredients [6,7]. These conventional cheese products set the standard for those sensory and functional qualities that plant-based cheese analogue (PCBA) product alternatives need to replicate if they are to be successful in any marketplace, especially in the Irish context, where cheese and dairy product manufacturing and consumption are so high.

Plant-based cheese analogues use water, fats, proteins, emulsifiers, hydrocolloids, and flavouring agents in an attempt to mimic conventional cheese products [8,9]. Such plant-based products typically have lower protein content, higher saturated fat and carbohydrate content, and different structural properties compared to established dairy products, complicating efforts to mimic the texture and melting characteristics of traditional cheese [10,11]. For example, plant proteins, with their larger molecular size and more complex quaternary structures, struggle to form the compact gel networks that casein proteins do, making it difficult to replicate functional properties such as meltability and flowability in plant-based cheeses [12].

Sensory attributes such as flavour and texture play a crucial role in determining consumer acceptance of cheese products. Traditional dairy cheeses are highly valued for their ability to deliver these qualities. However, consumer preference remains a significant challenge for plant-based products, primarily due to their often-unpleasant intrinsic flavours and odours, which can limit their appeal. These off-flavours, frequently described as “green,” “grassy,” and “beany”, arise from common volatile off-flavour (such as aldehydes, furans, alcohols, pyrazines, and ketones) as well as non-volatile compounds (like phenolics, peptides, alkaloids, and saponins), negatively impacting their consumer acceptance [13,14]. Assessing sensory properties is therefore essential for evaluating product performance and guiding the development of plant-based cheese products to overcome these challenges and meet consumer expectations [15].

There are different formats of cheese products, including block-style and slice-style, available in the market. Block cheeses are typically used for slicing and grating, while sliced cheeses are primarily used in sandwiches and burgers, where texture and meltability are critical. The future development of plant-based cheese analogue (PBCA) products must consider these traditional usage contexts, as consumers will expect PBCA formats to replicate the functional performance of their dairy-based counterparts. The objective of this study was to be the first comprehensive compositional, physicochemical, microstructural, and sensory benchmarking exercise of its kind in Ireland to assess commercially available, both block-style and slice-style, PBCA product formats. Unlike prior studies, which have focussed on single-format cheeses or placed emphasis on limited parameters, this work applies a multi-dimensional assessment approach, encompassing proximate analysis, colour changes during melting, confocal microscopy, rheology, thermal transitions (DSC), and sensory profiling using APLSR, to identify critical performance gaps in flavour, meltability, and texture. By systematically linking structural deficits (e.g., absence of protein–fat matrices and starch-driven rigidity) to negative sensory outcomes, this study highlights format-specific limitations and provides a roadmap for formulation improvements in next-generation PBCA products. It also provides a unique and independent perspective on Irish attitudes to current PBCA product formats on offer in a dairy-based marketplace.

## 2. Materials and Methods

### 2.1. Commercial Products

Five block-style and seven slice-style plant-based cheese analogues were identified through a survey of Irish retailers, including Aldi, Dunnes Stores, Lidl, Quay Co-op, Supervalu, and Tesco. These products were selected to represent a cross-section of popular brands and formulations available to Irish consumers, with a focus on those marketed as dairy-free cheese alternatives. To provide meaningful comparisons, two cheddar cheeses and two processed cheese products were included as dairy-based benchmarks, reflecting typical products used for slicing, grating, or melting in everyday culinary applications. Products were sourced from Irish retailers and subsequently stored at 4 °C and analysed within seven days of purchase. The details and ingredients listing from the product label for all experimental samples are presented in Table 1 (block-style products) and Table 2 (sliced products).

### 2.2. Compositional Analysis, pH, and Water Activity Measurements

The composition of all experimental samples used in this study was initially determined. The Kjeldahl method 2001.14 [16] was used to quantify the protein level and a nitrogen-to-protein conversion factor of 6.38 for cheddar and processed cheeses and 6.25 for plant-based products [17]. Fat content was determined using the Gerber method 933.05 for dairy [18] and the Soxhlet method 30-25.01 [19] employing Sox-Cap and Sox-Tec units (Foss UK Ltd., Runcorn, UK) for plant-based products. Moisture and ash contents were analysed by oven-drying method 926.08 (103 °C, 5 h) and incineration method 935.42 (800 °C, 5 h) [20,21]. Total carbohydrate content was calculated by subtracting the sum of protein, fat, ash, and moisture from 100. To determine the pH, 10 g of the sample was combined with 10 mL of water at ambient temperature and homogenized for 5 min using a Stomacher blender (Seward Ltd., Worthing, UK). Water activity was measured at 20 °C using a calibrated water activity meter (Aqua Lab, Decagon Devices, Pullman, WA, USA). Samples were prepared for water activity measurement by cutting cylinders of 5 mm height and 40 mm diameter prepared with a circular cutter and a meat slicer (Scharfen G330F., Witten, Germany) before analysis.

### 2.3. Colour Measurement

Sample colour (L*, a*, b*) was assessed using a CR400 chromameter (Konica Minolta, Tokyo, Japan), calibrated with a white tile, following the method outlined in [22]. Measurements were taken both before and after meltability tests were conducted (Section 2.5).

### 2.4. Confocal Laser Scanning Microscopy

Microstructure was examined using a confocal laser scanning microscope (OLYMPUS FV1000, Olympus Corporation, Tokyo, Japan). The staining procedure was adapted from a previously published method [23], where Nile Red was used to label fat and Fast Green FCF was used to label protein. A staining solution was prepared by mixing 600 μL of Nile Red (0.1 g/L in 1,2-propanediol) with 200 μL of Fast Green FCF (0.1 g/L) in water. Around 50 μL of this solution was applied to each sample, which was then stored at 4 °C for 10 min before imaging. The dyes were excited at 488 nm (Nile Red) and 633 nm (Fast Green FCF) [24], and images were acquired using a 20× objective lens.

### 2.5. Schreiber Meltability Test

The meltability of the products was evaluated using a modified version of the Schreiber test, based on the method described in [25]. Cylindrical samples measuring 5 mm in height and 40 mm in diameter were prepared. For block-style cheeses, samples were cut using a meat slicer and circular cutter. For slice-style cheeses, which are typically thinner, two slices were stacked to achieve the required height, and the same circular cutter was used to ensure uniform diameter. Samples were then kept at 4 °C until testing. Samples were kept in a covered glass Petri dish and heated at 232 °C for 5 min in an oven (Memmert, Schwabach, Germany). After heating, the samples were allowed to cool at room temperature for 30 min. Specimen expansion was assessed by measuring along six radial lines marked on concentric circles using a ruler. The meltability was calculated as the mean of these six measurements and reported as the percentage increase in sample size [26]. Images were captured both prior to and following the melting process.

### 2.6. Rheological Properties

#### 2.6.1. Dynamic Low Amplitude Oscillatory Shear Rheology

A controlled-stress rheometer (AR-G2, TA Instruments Ltd., Delaware, UK) with stainless steel parallel plates featuring a crosshatched surface was used to assess the rheological properties of the products, as detailed in [27]. Samples, measuring 2 mm in height and 50 mm in diameter, were prepared with the help of a cutter and meat slicer, then kept overnight at 4 °C. Before analysis, samples were allowed to equilibrate to room temperature. The analysis involved applying a shear strain of 0.08% at a frequency of 1 Hz while gradually increasing the temperature from 20 to 90 °C at a rate of 3 °C per min. The measurements included the storage modulus (G′), loss modulus (G″), loss tangent (Tan δ), and maximum loss tangent (Tan δ max). RheoCompass™ software (Version 1.30.1227 was used to process data.

#### 2.6.2. Texture Profile Analysis

A texture analyser (TA-XT2i, Stable Micro Systems, Godalming, UK) was used to conduct texture profile analysis (TPA) for the block-style products following the method outlined in [28]. Cylindrical test specimens (20 mm in diameter, 12 mm in height) were cut using a stainless-steel wire and a circular blade, then stored at 4 °C overnight. On the day of testing, samples were compressed to 25% of their initial height with a cylindrical probe of 50 mm in diameter using a two-cycle compression at a speed of 1.0 mm/s. The resulting textural parameters, including hardness, adhesiveness, springiness, and cohesiveness, were recorded for each product. [29].

#### 2.6.3. Uniaxial Compression Testing

Uniaxial compression testing was conducted on slice-style cheese analogues using a TAXT2i texture analyser (Stable Micro Systems Ltd., Godalming, UK), equipped with a P/6 cylindrical probe of diameter 6 mm. A cheese slice of 2 mm thickness was placed on the Texture Analyser Heavy Duty Platform (HDP/90) with an attachment in the form of a flat plate with a hole (9 mm diameter) cut out at the center to allow the full penetration of the probe through both the sample and the platform underneath. The test was performed at a compression rate of 1.0 mm/s for 6 mm, ensuring complete fracture and penetration through the 2 mm thick cheese sample. Hardness was reported for all samples.

### 2.7. Differential Scanning Calorimetry

Thermal characteristics of the samples were assessed using a differential scanning calorimeter (DSC821, Mettler-Toledo, Greifensee, Switzerland), which was equipped with liquid nitrogen cooling. A slightly modified method based on [27] was applied. Samples weighing between 12 and 18 mg were prepared, sealed in standard aluminum pans (40 µL, ME-26763, Mettler Toledo, Greifensee, Switzerland), and hermetically closed. Calibration of the equipment was performed for both temperature and heat flow using indium. The thermal transitions were examined by scanning the samples from −40 °C to 90 °C at a rate of 5 °C per minute. Resulting DSC profiles were analysed using the Mettler-Toledo STARe software (version 8.10).

### 2.8. Sensory Analysis

Sensory evaluation was conducted with 25 assessors (*n* = 25) recruited from the UCC student and staff community and included 8 males and 17 females across a range of age groups (25–60+ years) and nationalities (Irish (16), European (8), and Asian (1)). While panelists represented a variety of cultural backgrounds, all were residents of Ireland at the time of testing. Inclusion criteria specified that participants must be healthy, not following any medically prescribed diets, free from food allergies or swallowing issues, and regular consumers of cheese in general (not limited to cheddar). The study was approved by the UCC Social Research Ethics Committee (2023-142) and conducted in accordance with the Declaration of Helsinki. Informed consent was obtained from all participants.

The evaluations took place in the sensory laboratory at University College Cork, equipped with individual sensory booths under control conditions ISO 8589:2007, ISO 11136:2014 [30,31]. Each product sample was presented in 5–6 g portions. Slice-format products were served as is, and block-format samples were hand-sliced to match the same weight. All samples were coded with random three-digit numbers using RedJade (2025) sensory software and presented simultaneously in randomized order using a balanced incomplete block design to minimize bias. All samples were presented in duplicate [32,33] for both SAT and ODP as described below.

The sensory evaluation was carried out in a single session, divided into two parts. First, a sensory acceptance test (SAT) [32,33] was conducted with the 25 untrained panel members [34,35] using an unstructured line scale anchored at 0 (extremely dislike) and 10 (extremely like). The attributes evaluated included liking of appearance, aroma, flavour, texture, and overall acceptability.

In the second part of the session, participants (*n* = 2025) completed a modified Optimized Descriptive Profiling (ODP) [36,37,38,39]. A lexicon developed by an in-house expert panel (*n* = 10), with many years of cheese tasting experience and guided by vocabulary used in prior cheese sensory studies [34,35], was presented to the experimental panel. The descriptors used were described as obvious and unambiguous so as not to challenge the panel too much during subsequent training where descriptors were described and assessors were asked to taste them in each of the experimental products. The panel can be described as semi-trained with this method and used in an exploratory capacity. The cheese colour was assessed first (ivory-yellow), and then the following 16 attributes were assessed on a 10 cm line scale anchored from none to extreme: cheddar aroma, firmness in the mouth, pasty texture, crumbly texture, sweetness, saltiness, sourness, bitterness, cream flavour, cheddar flavour, dairy sweetness, dairy fat flavour, fruity (ester) flavour, off-flavour, and astringent aftertaste. Sensory data were processed and analysed using Unscrambler^®^ software (version 10.3) The nine cheese samples were presented simultaneously in a randomized and balanced order to each assessor to minimize first-order and carryover effects and bias [40]. Assessors could re-taste samples if they wished. Assessors were instructed to cleanse their palate with water and unsalted crackers between each sample.

### 2.9. Statistical Data Analysis

Experiments were conducted with nine replicates per product, derived from three independently purchased packages of each cheese analogue, with three subsamples taken from each package. Levene’s test was employed to assess the homogeneity of variance, and one-way analysis of variance (ANOVA) was performed using SPSS version 28 (SPSS Inc., Chicago, IL, USA). To identify statistically significant differences (*p* < 0.05) between the mean values of different samples, Tukey’s post hoc test was applied at a 95% confidence level. Data are expressed as mean ± standard deviation, with significant differences represented using superscript letters. Unscrambler X software, version 10.3 (CAMO ASA, Trondheim, Norway), was used for ANOVA-PSLR (APLSR) analysis of sensory data to process the raw data accumulated from the 25 test subjects. The X [1] matrix was designed as 0/1 design variables for treatment. The Y-matrix was designed for sensory variables. The optimal number of components in the APLSR models presented was determined to be 4 principal components. PC 1 versus PC 2 is presented; the other PCs did not yield additional information or provide any predictive improvement in the Y-matrix obtained through their examination. To derive significance indicators for the relationships determined in the quantitative APLSR, regression coefficients were analysed by jackknifing, which is based on cross-validation and stability plots [41]. The model explained calibrated and validated variances, which were Factor 1 (17%; 11%) and Factor 2 (17%; 5%). These relatively low values likely reflect the wide variation in product formulations. Additionally, hedonic analysis is for noisy data where wide variance can be anticipated. Also, the semi-trained panel will have more variance than a QDA panel. The cultural diversity of assessors, while representative of the Irish consumer population, may also have introduced perceptual variation due to differing sensory expectations. These factors represent some limitations to the study.

## 3. Results and Discussion

### 3.1. Formulation, Chemical Composition, pH, and Water Activity of Products

The formulations of the products are outlined in Table 1 and Table 2. Cheddar cheese had the simplest dairy-based formulation, while processed cheese comprised 60% cheese, along with modified starch, emulsifying salts, milk protein, and vegetable oils. In contrast, all vegan variants were primarily formulated using coconut oil and starch blends, with some including additional oils like shea. Only Plant 6 (slice format) contained shea oil in addition to coconut oil. Detailed measurements of composition, water activity and pH for block and slice formats are shown in Table 3 (Block) and Table 4 (Slice). The chemical composition and pH values of the cheddar and processed cheeses were consistent with values previously reported in the literature [42,43].

Among the block-style samples, cheddar cheese exhibited the highest levels of both protein (25.6 g/100 g) and fat (34.8 g/100 g), followed by the processed variant, which contained 18.5 g/100 g protein and 24.9 g/100 g fat. In contrast, protein content in plant-based alternatives was substantially lower, ranging from 0.1 to 1.7 g/100 g. The highest protein level among these was observed in Plant 4, likely due to the inclusion of potato protein, with Plant 1 following due to its lentil protein content. Moisture content peaked in Plant 4 (56.9 g/100 g), with Plant 2 at 52.2 g/100 g, while cheddar recorded the lowest value at 38.0 g/100 g. Ash content was highest in processed cheese (4.5 g/100 g). Regarding pH, plant-based samples ranged from 3.56 to 4.20, whereas cheddar and processed cheeses displayed notably higher values (5.28, 6.03).

Cheddar cheese had the lowest water activity among the products tested and was comparable to the mature cheddar results [44] and the retail cheddar cheese [42,45]. The processed cheese had a water activity of 0.98, which was consistent with typical processed cheese products exhibiting 50 g/100 g moisture (0.97–0.98) [46]. Plant 1, 2, and 4 products had water activity comparable to processed cheese.

Similarly, slice-style (Table 4) dairy cheese exhibited the highest protein content, with Plant 7 having the highest among others. This discrepancy is due to the high concentration of casein in dairy cheese [47]. The highest fat concentration was found in dairy cheese, while among plant-based cheeses, Plant 6 had the highest fat content, reflecting the use of fat sources like coconut and shea oils [48]. Compared to dairy cheese, moisture content was higher in plant-based cheeses due to the hydrocolloids and water-binding compounds present [10]. Ash content was lowest in Plant 7 but highest in processed cheese, indicating higher mineral levels. While cheddar cheese had no carbohydrates, plant-based cheeses varied, with Plant 7 being highest, linked to the presence of starches and carbohydrates [49,50]. Processed cheese had the highest pH, while the most acidic plant-based cheese was Plant 4 (4.7 ± 0.0). All samples had slightly different water activities; dairy-based cheese had the lowest (0.95 ± 0.0), while Plant 5 and Plant 6 had the highest values (0.98 ± 0.0).

### 3.2. Colour Characteristics

In the context of appearance of food, colour is crucial since it reflects quality factors like composition, consumer preference, and deterioration [51]. Processing conditions, pH, composition, age, use of colouring ingredients, and temperature all significantly impact cheese colour [52,53,54,55]. Given that temperature affects colour, measurements were made both before and after cheese-melting treatments using the CIELAB coordinates shown in Table 5 and Table 6.

In the case of block-style (Table 5) cheeses prior to melting, the highest L* value (brightness) was determined for Plant 4 (93.6), followed by Plant 5 (92.1) and Plant 3 (90.0), with Plant 2 possessing the lowest values (79.8). Most samples possessed negative a* values (redness to greenness), except for processed cheese (9.1) and Plant 2 (10.3), indicating a redder hue. Positive b* values (yellowness) were observed, with Plant 2 possessing the highest (50.6) values, followed by processed cheese (47.6), and with Plant 4 having the lowest (7.7) values. Increased a* and b* values in processed cheese and Plant 2 may have resulted from the addition of colouring food ingredients like paprika extract and β-carotene [56,57]. After melting, significant changes in colour parameters were observed due to phase change, fat loss, moisture evaporation, and protein matrix collapse [58]. The L* values decreased across all samples, indicating reduced brightness. The a* values generally decreased, except for Plant 2, which presented an increase to 15.49, thereby maintaining its high redness values. The b* values increased in most samples, indicating greater yellowness after melting, with Plant 2 showing the highest increase (68.6) in these values. These changes can be attributed to modifications in translucency during melting [59].

Slice-style cheeses (Table 6), before melting, generally exhibited higher lightness (L*) values, indicating a lighter colour compared to dairy cheese, with Plant 1 and Plant 4 being the lightest in appearance. Most other samples, including dairy cheese, had negative a* values, indicating greenish hue, whereas processed cheese, Plant 2 and Plant 6, displayed positive a* values (reddish hue due to added colourants). The highest b* values were observed in processed cheese, and Plant 2 and Plant 6 presented a strong yellow hue. After melting, all samples showed a decrease in lightness, with Plant 2 and Plant 6 presenting the most significant darkening. The a* values became more negative in most samples, indicating a shift towards a greener hue, except for processed cheese, Plant 2, and Plant 6, which conversely became redder in appearance. The b* values increased for all samples post-melting.

### 3.3. Microstructure Characteristics

**Figure 1:**
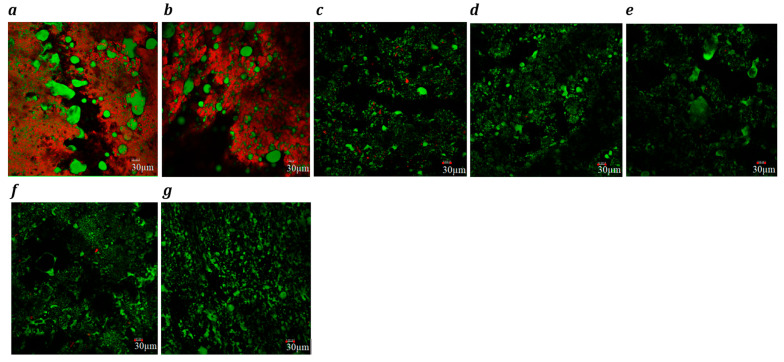
Confocal laser scanning microscopy images (×20) of block-style cheddar (**a**) processed (**b**), Plant 1 (**c**), Plant 2 (**d**), Plant 3 (**e**), Plant 4 (**f**) and Plant 5 (**g**) products. Fat globules are stained green, and protein is stained red. Each image includes a 30 µm scale bar.

**Figure 2:**
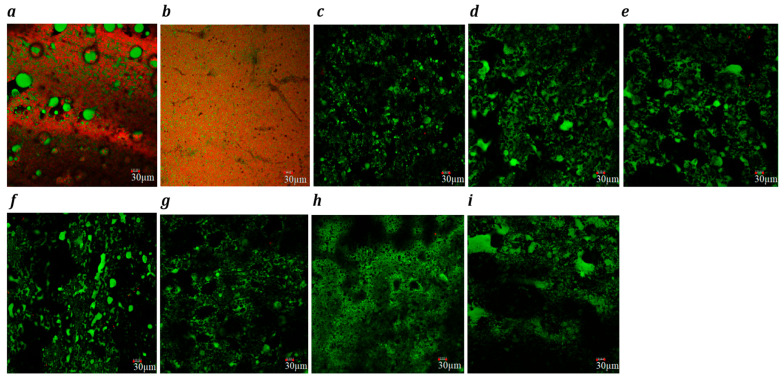
Confocal laser scanning microscopy images (×20) of slice-style cheddar, (**a**) processed (**b**), Plant 1 (**c**), Plant 2 (**d**), Plant 3 (**e**), Plant 4 (**f**), Plant 5 (**g**), Plant 6 (**h**) and Plant 7 (**i**) products. Fat globules are stained green, and protein is stained red. Each image includes a 30 µm scale bar.

The confocal microscopy images (Figure 1, block-style, and Figure 2, slice-style) revealed significant differences in the microstructural organization of proteins (red) and fats (green) among dairy, processed cheese, and plant-based cheese analogues.

Analysis of the microstructure of block-style cheeses showed that cheddar cheese (Figure 1a) exhibited predominantly large spherical coalesced fat pools embedded within a continuous protein matrix, aligning with earlier findings [60,61]. In processed cheese (Figure 1b), both spherical and irregularly shaped fat globules, along with starch granules (visible as black zones), were uniformly distributed, forming a consistent fat–protein network. However, the uniform distribution of fat and protein with no distinct granules suggests that starch was fully gelatinized and integrated into the matrix [62]. The microstructure was comparable to commercial processed cheeses [62]. Plant-based products also exhibited non-spherical fat globules within a starch–hydrocolloid matrix. The absence of distinct starch granules indicated full gelatinization. The Plant 5 product showed the smallest, densely packed fat globules (Figure 1g), while Plant 3 had significantly larger ones compared to the other plant-based samples (Figure 1e). Protein staining in most plant-based cheeses was sparse and patchy, reflecting their low protein content (0.1–1.7 g/100 g) and poor network formation. Notably, Plant 1 (Figure 1c) and Plant 4 (Figure 1f) showed slightly more red staining, but the protein remained unevenly dispersed, lacking the continuous matrix seen in dairy cheese. However, both exhibited sparse and unevenly distributed protein aggregates within the cheese matrix, indicating a lack of homogenous protein network formation.

For slice-style cheddar cheese (Figure 2a), protein was shown to form a continuous network with larger, dispersed fat globules (green), providing elasticity and desirable melting properties [63]. Processed cheese showed a fragmented protein matrix and smaller, finely dispersed fat globules due to emulsifying salts and heating that created a homogeneous and stable product [64,65].

Plant-based cheeses exhibited sparse red dots, indicating low protein content. Plant 1 and Plant 4 had small, well-dispersed fat globules. Plant 6 presented a very fine distribution of fat globules and intricate structure of starch, indicative of the use of emulsifying agents similar to processed cheese [7]. Plant 2 had larger, uneven fat globules, while Plants 3 and 5 possessed moderately sized fat globules. Plant 7 possessed mixed-size fat globules.

### 3.4. Meltability

**Figure 3:**
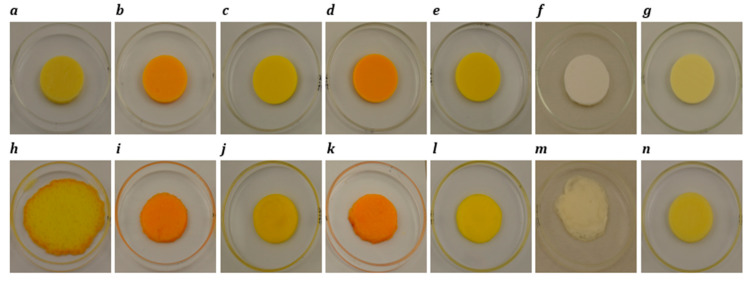
Photographs of block-style cheddar, processed, Plant 1, Plant 2, Plant 3, Plant 4 and Plant 5 products before (**a**–**g**) and after (**h**–**n**) oven-melting at 232 °C for 5 min.

The results of the Schreiber test for meltability are reported in Table 3 and Table 4. For block-style cheeses, when heated at 232 °C for 5 min (Figure 3h), cheddar cheese demonstrated the highest expansion in diameter (81.7%), significantly outperforming plant-based samples, which ranged from 0.0% to 17.2%. Melting occurred due to fat flow and moisture evaporation, stretching the protein network. Fat acted as a lubricant, aiding protein mobility at higher temperatures [58]. The processed cheese (2.2%) had the lowest meltability, likely due to the formulation’s starch content. The disruption of the protein matrix by swollen starch granules contributed to poor melting behaviour [66]. Additionally, the presence of emulsifying salts was found to negatively affect melting performance in processed cheese [67]. Starch and hydrocolloids in plant-based products formed a continuous network, hindering melting due to starch gelatinization properties [54]. Among vegan products, Plant 5 (Figure 3n) showed no melting, and the remainder of plant-based products presented diameter expansions between 2.2 and 17.2%. Plant 4 exhibited the highest melting among plant-based products (17.2%), and this was attributed to its higher moisture content.

**Figure 4:**
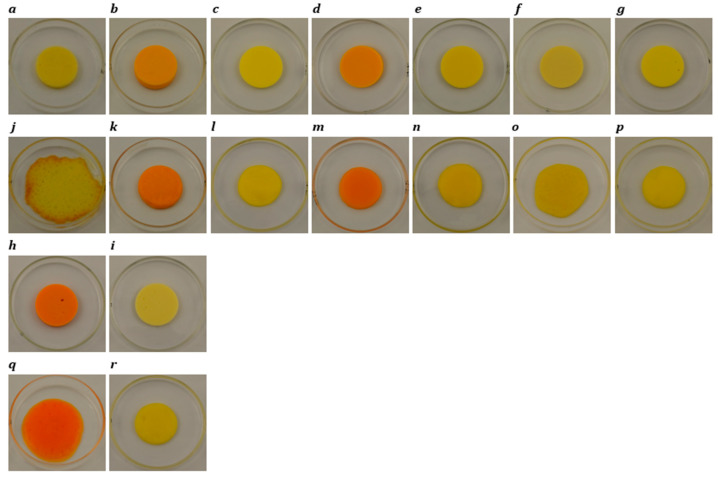
Photographs of slice-style cheddar, processed, Plant 1, Plant 2, Plant 3, Plant 4, Plant 5, Plant 6, and Plant 7 products before (**a**–**i**) and after (**j**–**r**) oven-melting at 232 °C for 5 min.

Similarly, in slice-style products (Figure 4), cheddar cheese exhibited the highest meltability, attributed to its well-developed protein network and larger, uniformly distributed fat globules, which facilitated better flow and spread upon heating [63]. In contrast, processed cheese showed low meltability. Among the plant-based cheeses, most samples (Plants 1, 2, 3, 5, and 7) also demonstrated low meltability owing to the potential use of stabilizers and thickeners that inhibited melting [68]. However, Plant 4 (34.5%) and Plant 6 (46.1%) exhibited significantly higher meltability, which could be attributed to their higher fat contents.

### 3.5. Rheological Properties

#### 3.5.1. Viscoelastic Properties of Cheese Samples

**Figure 5:**
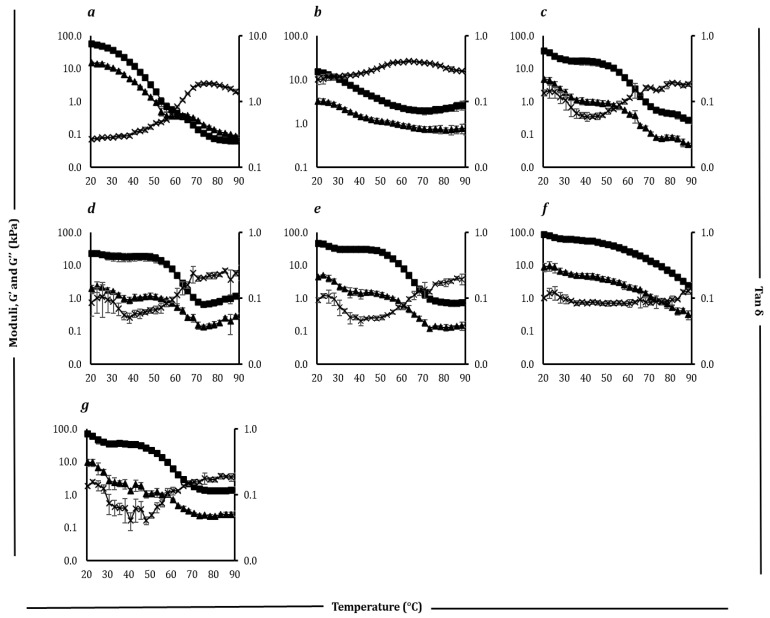
Rheological profiles showing storage modulus (G′) (filled square), loss modulus (G″) (filled triangle), and loss tangent (Tan δ) (cross) as a function of temperature (20–90 °C) for block style cheddar (**a**), processed (**b**), Plant 1 (**c**), Plant 2 (**d**), Plant 3 (**e**), Plant 4 (**f**), and Plant 5 (**g**) products.

**Figure 6:**
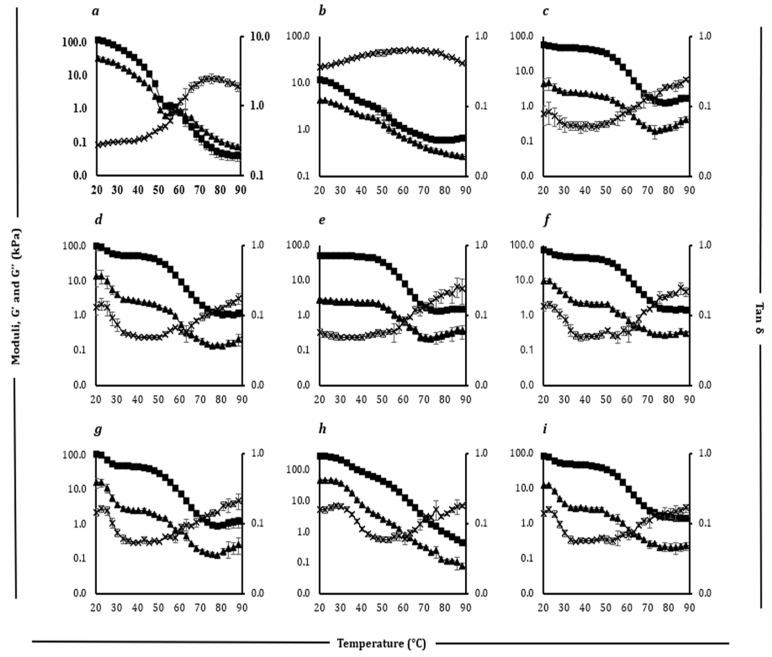
Rheological profiles showing storage modulus (G′) (filled square), loss modulus (G″) (filled triangle), and loss tangent (Tan δ) (cross) as a function of temperature (20–90 °C) for slice-style cheddar (**a**), processed (**b**), Plant 1 (**c**), Plant 2 (**d**), Plant 3 (**e**), Plant 4 (**f**), Plant 5 (**g**), Plant 6 (**h**), and Plant 7 (**i**) products.

The rheological behaviour of the samples was evaluated using dynamic low-amplitude oscillatory shear tests, with results illustrated in Figure 5 and Figure 6. Detailed values for the key parameters are presented in Table 7 and Table 8.

For block-style (Table 7) cheddar cheese, G′ (elastic modulus) and G″ (viscous modulus) decreased with increasing temperature. Initially, G′ (56.9) exceeded G″ (15.1), reflecting solid-like behaviour. Both moduli sharply declined at 40–50 °C due to fat melting and protein matrix disruption, leading to structural breakdown and desirable melting properties [6]. The tan δ (G″/G′) increased, indicating a shift from solid-like (tan δ < 1) to fluid-like (tan δ > 1) behaviour, with a peak (1.9) at 73.3 °C, reflecting maximum fluidity. After the peak, tan δ decreased slightly due to hydrophobic protein interactions [69]. Cheddar cheese′s rheological profile matched previous reporting for aged cheese [70], with G′ and G″ crossing over (0.36 kPa) at 62.8 °C, indicating the transition to fluid-like behaviour. This rheological behaviour aligns with its melting characteristics (Section 3.4).

Processed cheese exhibited a gradual decrease in moduli and a smoother tan δ increase, reflecting stability due to emulsifiers and stabilizers [71]. Among plant-based samples and processed cheese, the maximum loss tangent (Tan δ_max_) values fell between 0.1 and 0.3, indicating minimal or no meltability. This behaviour was consistent with the limited fluid transition observed, likely resulting from the presence of starch and hydrocolloids that restrict melting, an effect also documented in prior studies [72].

Slice-style cheddar and processed cheeses showed similar rheological patterns to block styles. All slice-style plant-based products exhibited high elasticity at elevated temperatures, attributed to starch content, with slight softening above 50 °C due to starch gelatinization [73]. The exceptional behaviour of Plant 6 (Figure 6h) might be due to the combination (shea and coconut oil) and amount (32 g/100 g) of fats used.

#### 3.5.2. Textural Properties of Block Style Products

Textural properties, including cohesiveness, adhesiveness, hardness, and springiness for block-type products, are shown in Table 7. Dairy cheese exhibited the highest hardness, moderate springiness, and cohesiveness, attributed to a firm and resilient natural protein and fat matrix, while the hardness value for the cheddar cheese was in line with the previous study [28]. Processed cheese exhibited lower hardness and springiness but higher adhesiveness, due to emulsifiers and stabilizers. Plant-based cheeses showed wide variability in hardness, with Plant 2 and Plant 3 being hardest, while Plant 4 was the softest. Adhesiveness was generally lower, with Plant 1 being the least adhesive and Plant 5 being the most adhesive. Plant 2 demonstrated the highest springiness and cohesiveness, indicating effective hydrocolloid use for a resilient texture like dairy cheese.

These results underscore the role of fats, hydrocolloids, and texturizing agents in achieving dairy-like textures in plant-based cheese while addressing variability in formulations [6,67,74].

#### 3.5.3. Hardness of Slice Style Products

Uniaxial compression testing is a fundamental method used to measure the mechanical properties of materials. Hardness values of slice-style products (Table 8) showed dairy cheese as having a moderate hardness, attributed to its balanced protein and fat matrix. Processed cheese had the lowest hardness due to emulsifiers and stabilizers [75,76]. Among the plant-based cheeses, hardness varied widely, with Plant 6 being the firmest, which is likely due to the high proportion of proteins present in the sample or hydrocolloids, and Plant 4 also shows high firmness, suggesting strong structural integrity. In contrast, Plant 1 (2.8 ± 0.03 N) and Plant 3 (3.3 ± 0.02 N) had hardness values closer to dairy cheese, indicating that product formulations focussed on balancing firmness and elasticity, similar to natural cheese [77]. Plant 7 (3.8 ± 0.02 N) was firmer than dairy-based cheese but softer than the firmest plant-based samples.

### 3.6. Thermal Behaviour of Products

Differential scanning calorimetry (DSC) characterizes the thermal behaviour of dairy-based cheese, processed cheese, and other plant-based cheese substitutes, and the results are summarized in Table 7 and Table 8.

The block-style dairy-based products exhibited two distinct endothermic peaks, indicative of multi-component fat systems (Table 7). In cheddar, the first peak occurred at 13.8 °C, and the second at 30.1 °C, which correspond to the melting of low/middle melting fractions (LMF/MMF) and high melting fractions (HMF) of milk fat, respectively [62]. The cheddar cheese thermogram matched the findings from earlier studies [78,79], which demonstrated a constant melting pattern. Processed cheese also exhibited two peaks due to its blend of natural cheeses and emulsifying salts, leading to variable melting temperatures [7]. For plant-based cheese analogues, most showed a single melting peak in the range of ~20–21 °C, which aligns with the melting point of coconut oil, the primary fat used in these formulations [80,81]. These values indicate a more homogeneous fat composition and a narrow melting range.

For slice-style cheese (Table 8), distinct peaks in dairy cheese also represented the melting of milk fat and protein transitions. Processed cheese had a melting profile, peaking at ~30 °C, reflecting palm oil melting and the emulsified fat-protein matrix [6].

Most plant-based cheeses, except Plant 6, showed peaks at ~20 °C, linked to coconut oil usage. Plant 6, with shea and coconut oils, displayed a peak near 30 °C. The absence of peaks at 60–80 °C suggests minimal starch gelatinization, likely due to pre-gelatinized starch use, which does not undergo typical transitions in DSC analysis [82]. Thermographs for block-style and slice-style products are presented in supplementary data (Figure S1, Figure S2, respectively). 

### 3.7. Sensory Profile Analysis

ANOVA-Partial Least Squares Regression (APLSR plot) illustrates the sensory profiles of cheeses, with Factor 1 (x-axis) and Factor 2 (y-axis) representing variance. Blue points denote products, and red points represent sensory attributes. Proximity between a product and an attribute indicates a positive association, while opposing positions imply a negative correlation.

**Figure 7:**
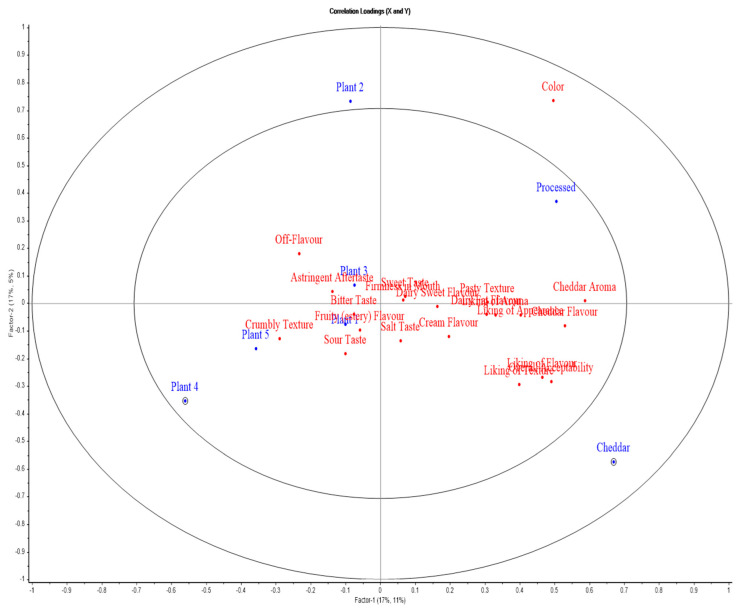
APLSR graph for sensory evaluation of block-style products. Plotted are cheese variants (blue) versus sensory (hedonic and descriptive) attributes (red).

Figure 7 presents the APLSR plot for block-style products. Cheddar cheese emerged as the most acceptable to assessors, followed by processed cheese, being strongly associated with positive attributes such as liking of appearance, liking of flavour, liking of aroma, liking of texture, overall acceptability, cheddar aroma, and cheddar flavour, which is consistent with the literature [6]. The processed product shows a unique profile, strongly associated with attributes like colour and cheddar aroma, likely due to its standardized formulation and controlled processing conditions, aligning with previous findings [83]. It is positioned away from the centre but to the right, indicating a product that may appeal in specific ways but lacks a broad sensory profile like cheddar. All plant-based cheeses were generally disliked and were close to undesirable attributes such as off-flavour, crumbly texture, astringent aftertaste, etc. Factor 1 and Factor 2 in this model explained 17% and 11% of the total variance, respectively (28% combined). These relatively low values likely reflect the wide variation in product formulations.

**Figure 8:**
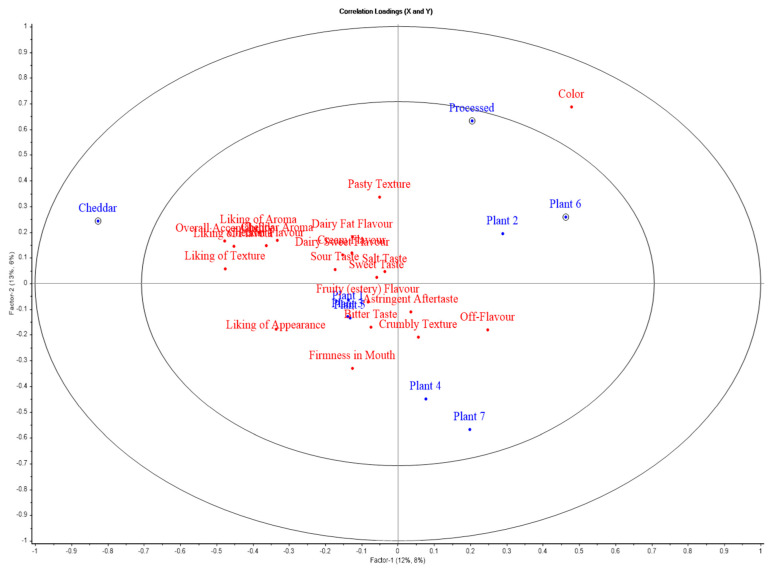
APLSR graph for sensory evaluation of slice-style products. Plotted are cheese variants (blue) versus sensory (hedonic and descriptive) attributes (red).

Beta coefficients for block-style products are presented in Table 9, which indicates the strength and direction of the relationship between each sensory attribute and the respective cheese product (reflecting the direction of correlation, + or −ve). Table 10 presents correlation values for each block-style product relative to sensory attributes (*p*-values are the significance of correlation), with green indicating positive significant correlations and red indicating negative significant correlations. Cheddar is consistently associated with positive sensory attributes, as evidenced by high beta coefficients for liking of appearance, liking of aroma, liking of flavour, liking of texture, overall acceptability, cheddar aroma, etc. Processed cheese was also liked by assessors after cheddar cheese, as it presented significant positive associations (*p* < 0.05) with attributes such as liking of flavour, colour, cheddar aroma, cheddar flavour, etc. On the other hand, all plant-based cheeses were disliked, with Plant 2 and Plant 5 being the most disliked, as itthey presented significant negative associations with attributes such as liking of flavour, liking of texture, overall acceptability, colour, and pasty texture. Plant 4 was positioned far from attributes such as appearance, colour, firmness in mouth, cheddar flavour, and aroma in the APLSR plot, indicating weak or negative sensory associations with these characteristics. Plants 1 and 3 were also negatively associated with desirable attributes. Moreover, all the plant-based products are positively associated (Table 9) with undesirable sensory characteristics such as crumbly texture, off flavour, and astringent aftertaste, highlighting their limitation in the replication of milk products. The sensory evaluation of slice-style cheeses is presented in the APLSR plot (Figure 8). Factor 1 and Factor 2 accounted for 12% and 8% of the total variance, respectively, explaining a combined 20% of the variation. Cheddar cheese is positioned to the far left of the plot for Factor 1 and slightly above the origin for Factor 2, indicating that it is distinct from the other products. Cheddar is closely associated with positive attributes such as liking of appearance, liking of aroma, liking of flavour, liking of texture, overall acceptability, cheddar flavour, etc. This positioning suggests that cheddar is perceived very positively, aligning with traditional expectations for this product type. Assessors did not like the processed cheese, especially due to its sticky and pasty texture, which could be attributed to its formulation process, which resulted in a more homogenized product. All the plant-based cheese products were disliked as they were far from desirable attributes and closer to undesirable sensory characteristics.

The combined results from Table 11 (beta coefficients) and Table 12 (correlation values) reinforce the sensory profiles suggested by the APLSR plot for slice-style products. Cheddar stands out with high positive beta coefficients and strong correlations across key sensory attributes such as liking of appearance, liking of aroma, liking of flavour, liking of texture, overall acceptability, cheddar aroma, cheddar flavour, and dairy fat flavour, confirming its favourable sensory profile. In contrast, processed cheese shows negative beta coefficients and correlations for most sensory attributes, particularly pasty texture, which could be the reason for its dislike. Plants 4, 6 and 7 were significantly negatively correlated with desirable attributes such as liking of aroma, liking of flavour, and overall acceptability but significantly positively associated with undesirable attributes like off flavour. Plants 1, 3 and 5 showed non-significant positive correlations with many attributes like liking of texture, liking of appearance, liking of flavour, and overall acceptability but also negative correlations for other attributes such as colour, crumbly texture, and off-flavour, indicating a mixed sensory profile that is reflected in their moderate placement in the APLSR plot. Lastly, Plant 2 is significantly negatively correlated with the liking of flavour, pasty texture, and sour taste. Negative attributes such as off-flavour, astringent aftertaste, and crumbly texture significantly detract from their acceptability, highlighting the sensory limitations of plant-based cheese alternatives. These findings emphasize the importance of ingredient selection and fermentation to improve the texture and flavour of plant-based cheeses [14,84].

## 4. Conclusions

This study provides the first Irish-based, multi-format benchmarking of plant-based cheese analogues against conventional dairy cheeses, revealing critical formulation gaps that hinder performance. Plant-based cheeses were found to have substantially lower protein levels and lacked the microstructural integrity, particularly protein–fat networks, necessary for desirable meltability and texture. These structural deficiencies were directly associated with poor sensory ratings, especially in flavour and texture, across both block- and slice-style formats. By linking compositional and microstructural limitations to functional and sensory outcomes, this research highlights the specific challenges PBCAs face in mimicking dairy cheese. Importantly, the integrated methodology, combining microscopy, thermal, and rheological assessments with advanced sensory profiling, offers a diagnostic framework not previously applied to commercial PBCAs. These findings offer clear direction for formulation improvements, optimized fat systems, and fermentation strategies to close the performance gap between plant-based and dairy cheeses. Future research should explore the role of microbial cultures, protein–starch interactions, and clean-label emulsifiers in enhancing structure, flavour, and meltability of plant-based cheese alternatives.

## Figures and Tables

**Table 1:** List of ingredients and images for block-style products.

Products	Ingredients	Pictures
Cheddar	Milk, salt, and rennet	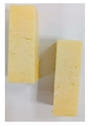
Processed	Cheese (60 g/100 g), modified maize starch, water, whey powder (milk), vegetable oils (coconut, palm), milk protein, tri-calcium phosphate, emulsifying salts (sodium phosphates, sodium polyphosphate), colour (paprika, carotene), acidity regulator (citric acid)	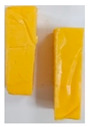
Plant 1	Water, coconut oil (24 g/100 g), modified starch, starch, sea salt, lentil protein, cheddar flavour, acidity regulator (lactic acid), olive extract, colour (beta-carotene), vitamin B12	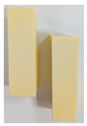
Plant 2	Water, modified starch, coconut oil (21 g/100 g), sea salt, starch, cheddar flavour, olive extract, vitamin B12, colour (beta-carotene, paprika extract)	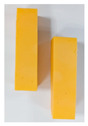
Plant 3	Water, modified maize starch, coconut oil (24 g/100 g), maize starch, modified potato starch, sea salt, modified tapioca starch, flavouring, vitamin B12, olive extract, colour (carotenes)	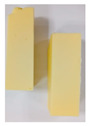
Plant 4	Water, potato protein, coconut oil (25 g/100 g) (non-hydrogenated), starch, sea salt, vegan flavourings, acidity regulator (lactic acid) (non-dairy), olive extract, vitamin B12	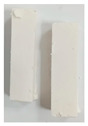
Plant 5	Water, coconut oil (24 g/100 g) (non-hydrogenated), modified starch, starch, tapioca maltodextrin, sea salt, vegan natural flavourings, olive extract, colour (natural beta-carotene), vitamin B12	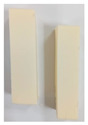

**Table 2:** List of ingredients and images for slice-style products.

Products	Ingredients	Pictures
Cheddar	Milk, rennet, salt	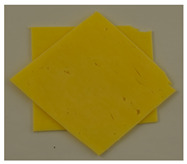
Processed	Cheese (60 g/100 g), water, milk protein, palm oil, modified potato starch, calcium phosphate, salt, salts (E452, E339), flavouring (milk), acidity regulator (lactic acid) (milk), natural colours (beta carotene, paprika extract)	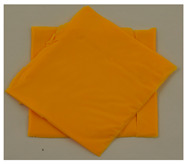
Plant 1	Water, coconut oil (23 g/100 g), modified starch, starch, sea salt, flavouring, olive extract, colour (beta-carotene), vitamin B12	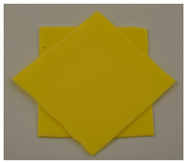
Plant 2	Water, coconut oil (23 g/100 g), modified starch, starch, sea salt, cheddar flavour, olive extract, colour (beta-carotene, paprika), vitamin B12	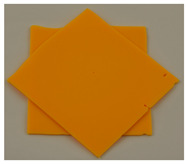
Plant 3	Water, coconut oil (23 g/100 g), modified starch, starch, sea salt, smoke flavour, olive extract, colour (beta-carotene), vitamin B12	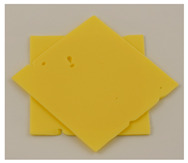
Plant 4	Water, coconut oil (25 g/100 g), modified potato starch, salt, calcium lactate, preservative (sorbic acid), natural flavouring, natural colour (beta-carotene), iron, vitamin D2, B6 & B12	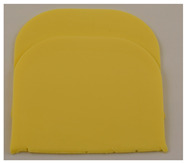
Plant 5	Water, coconut oil (24 g/100 g) (non-hydrogenated), modified starch, sea salt, olive extract, vegan flavourings, colour (natural beta-carotene), vitamin B12	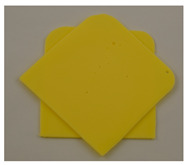
Plant 6	Water, shea oil, coconut oil, modified starch, salt, flavouring, lemon juice powder, spice extract, colour (beta-carotene)	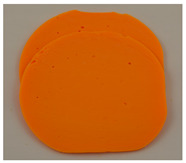
Plant 7	Water, coconut oil (24 g/100 g) (non-hydrogenated), modified starch, sea salt, olive extract, vegan flavourings, colour (natural beta-carotene), vitamin B12	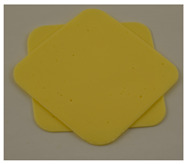

**Table 3:** Chemical composition, pH, water activity, and meltability of block-style variants.

	Cheddar	Processed	Plant 1	Plant 2	Plant 3	Plant 4	Plant 5
Protein (%)	25.6 ± 0.1 ^a^	18.5 ± 0.1 ^b^	1.2 ± 0.0 ^d^	0.1 ± 0.0 ^e^	0.1 ± 0.0 ^e^	1.7 ± 0.0 ^c^	0.1 ± 0.0 ^e^
Fat (%)	34.8 ± 0.8 ^a^	24.9 ± 0.2 ^c^	23.8 ± 0.2 ^d^	21.1 ± 0.2 ^f^	22.6 ± 0.4 ^e^	24.2 ± 0.8 ^cd^	26.6 ± 0.1 ^b^
Moisture (%)	38.0 ± 0.1 ^g^	47.8 ± 0.1 ^f^	50.7 ± 0.0 ^d^	53.2 ± 0.1 ^b^	51.9 ± 0.1 ^c^	56.9 ± 0.1 ^a^	46.9 ± 0.1 ^e^
Ash (%)	3.8 ± 0.0 ^b^	4.5 ± 0.0 ^a^	2.4 ± 0.1 ^c^	2.2 ± 0.1 ^d^	2.1 ± 0.0 ^d^	2.4 ± 0.0 ^c^	2.2 ± 0.0 ^d^
CHO * (%)	<0.0 ^e^	4.8 ± 0.2 ^d^	21.9 ± 0.3 ^b^	23.4 ± 0.2 ^a^	23.3 ± 0.4 ^ab^	14.9 ± 0.8 ^c^	24.2 ± 0.1 ^a^
pH	5.28 ± 0.0 ^b^	6.03 ± 0.0 ^a^	4.06 ± 0.0 ^d^	3.56 ± 0.0 ^e^	4.01 ± 0.0 ^d^	3.50 ± 0.0 ^f^	4.20 ± 0.0 ^c^
Water activity	0.95 ± 0.0 ^c^	0.98 ± 0.0 ^a^	0.98 ± 0.0 ^a^	0.98 ± 0.0 ^a^	0.96 ± 0.0 ^b^	0.98 ± 0.0 ^a^	0.96 ± 0.0 ^b^
Meltability (%)	81.7 ± 4.4 ^a^	2.2 ± 0.4 ^c^	6.7 ± 1.7 ^c^	2.2 ± 2.6 ^c^	3.3 ± 1.7 ^c^	17.2 ± 7.5 ^b^	0.0 ^d^

Values within the same row that carry distinct superscript letters (a–g) indicate statistically significant differences at *p* < 0.05. * CHO: Carbohydrates.

**Table 4:** Chemical composition, pH, water activity, and meltability of slice-style variants.

	Cheddar	Processed	Plant 1	Plant 2	Plant 3	Plant 4	Plant 5	Plant 6	Plant 7
Protein (%)	25.2 ± 0.4 ^a^	12.9 ± 0.0 ^b^	0.1 ± 0.0 ^c^	0.1 ± 0.0 ^c^	0.1 ± 0.0 ^c^	0.1 ± 0.0 ^c^	0.1 ± 0.0 ^c^	0.2 ± 0.0 ^c^	0.2 ± 0.0 ^c^
Fat (%)	35.0 ± 0.5 ^a^	20.5 ± 0.8 ^f^	22.5 ± 0.3 ^de^	22.7 ± 0.1 ^de^	21.6 ± 0.3 ^e^	25.3 ± 0.4 ^c^	22.9 ± 0.35 ^d^	32.0 ± 0.1 ^b^	25.8 ± 0.0 ^c^
Moisture (%)	37.5 ± 0.0 ^h^	53.4 ± 0.0 ^a^	52.9 ± 0.2 ^b^	52.4 ± 0.0 ^c^	52.6 ± 0.0 ^c^	48.2 ± 0.1 ^e^	51.8 ± 0.0 ^d^	42.7 ± 0.1 ^g^	47.3 ± 0.0 ^f^
Ash (%)	3.7 ± 0.0 ^b^	5.7 ± 0.0 ^a^	2.3 ± 0.0 ^d^	2.3 ± 0.0 ^d^	2.4 ± 0.0 ^d^	2.6 ± 0.1 ^c^	2.3 ± 0.1 ^d^	2.2 ± 0.1 ^d^	1.8 ± 0.0 ^e^
CHO * (%)	<0.0 ^e^	7.8 ± 0.8 ^d^	22.1 ± 0.5 ^c^	22.6 ± 0.1 ^c^	23.3 ± 0.4 ^bc^	23.8 ± 0.5 ^ab^	22.8 ± 0.4 ^bc^	22.9 ± 0.3 ^bc^	24.9 ± 0.1 ^a^
pH	5.32 ± 0.0 ^a^	6.00 ± 0.0 ^b^	4.10 ± 0.0 ^e^	4.05 ± 0.0 ^f^	3.91 ± 0.0 ^g^	4.71 ± 0.0 ^c^	3.89 ± 0.0 ^h^	4.49 ± 0.0 ^d^	4.47 ± 0.0 ^d^
Water activity	0.95 ± 0.0 ^d^	0.97 ± 0.0 ^b^	0.97 ± 0.0 ^b^	0.98 ± 0.0 ^a^	0.97 ± 0.0 ^b^	0.97 ± 0.0 ^b^	0.96 ± 0.0 ^c^	0.97 ± 0.0 ^b^	0.96 ± 0.0 ^c^
Meltability (%)	83.3 ± 3.3 ^a^	4.0 ± 0.0 ^d^	1.7 ± 0.0 ^d^	1.8 ± 0.2 ^d^	1.7 ± 0.0 ^d^	34.5 ± 2.6 ^c^	2.2 ± 0.9 ^c^	46.1 ± 1.9 ^b^	1.7 ± 0.0 ^c^

Values within the same row that carry distinct superscript letters (a–h) indicate statistically significant differences at *p* < 0.05. * CHO: Carbohydrates.

**Table 5:** Colour space values (L*, a*, b*) before and after melting for block-style products.

	Cheddar	Processed	Plant 1	Plant 2	Plant 3	Plant 4	Plant 5
**Before melting**							
L*	83.3 ± 0.2 ^e^	76.7 ± 1.1 ^g^	88.2 ± 0.1 ^d^	79.8 ± 0.4 ^f^	90.0 ± 0.1 ^c^	93.6 ± 0.4 ^a^	92.1 ± 0.1 ^b^
a*	−3.6 ± 0.1 ^f^	9.1 ± 0.2 ^b^	−1.6 ± 0.0 ^d^	10.3 ± 0.1 ^a^	−4.2 ± 0.0 ^g^	−1.3 ± 0.0 ^c^	−2.3 ± 0.0 ^e^
b*	28.6 ± 0.5 ^e^	47.6 ± 0.2 ^b^	29.5 ± 0.3 ^d^	50.6 ± 0.5 ^a^	34.5 ± 0.1 ^c^	7.7 ± 0.1 ^g^	16.5 ± 0.1 ^f^
**After melting**							
L*	73.7 ± 0.9 ^e^	67.5 ± 0.4 ^f^	77.7 ± 0.4 ^d^	68.2 ± 4.1 ^b^	78.7 ± 0.9 ^c^	88.5 ± 0.3 ^a^	81.9 ± 0.5 ^b^
a*	−6.3 ± 0.1 ^g^	8.8 ± 0.4 ^b^	−1.9 ± 0.1 ^d^	15.5 ± 0.2 ^a^	−5.8 ± 0.1 ^f^	−1.2 ± 0.0 ^c^	−4.2 ± 0.0 ^e^
b*	27.8 ± 0.6 ^d^	52.3 ± 0.2 ^b^	51.1 ± 0.5 ^c^	68.6 ± 2.0 ^a^	53.8 ± 0.7 ^b^	11.3 ± 0.2 ^f^	22.3 ± 0.1 ^e^

Values within the same row that carry distinct superscript letters (a–g) indicate statistically significant differences at *p* < 0.05.

**Table 6:** Colour space values (L*, a*, b*) before and after melting for slice-style products.

	Cheddar	Processed	Plant 1	Plant 2	Plant 3	Plant 4	Plant 5	Plant 6	Plant 7
**Before melting**									
L*	81.7 ± 0.4 ^f^	80.6 ± 0.3 ^g^	86.9 ± 0.1 ^c^	79.2 ± 0.1 ^h^	85.8 ± 0.1 ^e^	88.1 ± 0.0 ^a^	87.4 ± 0.1 ^b^	75.4 ± 0.2 ^i^	86.7 ± 0.1 ^d^
a*	−3.9 ± 0.01 ^e^	11.4 ± 0.2 ^b^	−5.2 ± 0.0 ^h^	9.0 ± 0.1 ^c^	−4.1 ± 0.1 ^f^	−3.0 ±0.0 ^d^	−4.9 ± 0.1 ^g^	17.5 ± 0.1 ^a^	−3.9 ± 0.0 ^e^
b*	30.4 ± 0.3 ^f^	45.9 ± 0.2 ^b^	35.8 ± 0.1 ^d^	51.5 ± 0.1 ^a^	35.6 ± 0.3 ^d^	32.4 ± 0.0 ^f^	34.6 ± 0.1 ^e^	44.5 ± 0.8 ^c^	23.9 ± 0.0 ^g^
**After melting**									
L*	70.9 ± 0.6 ^e^	77.5 ± 0.8 ^b^	76.6 ± 1.2 ^bc^	63.7 ± 0.0 ^f^	75.9 ± 0.1 ^c^	81.5 ± 1.1 ^a^	73.8 ± 0.3 ^d^	64.5 ± 0.4 ^f^	70.4 ± 0.0 ^e^
a*	−6.4 ± 0.0 ^g^	17.8 ± 0.2 ^b^	−5.4 ± 0.1 ^f^	15.0 ± 0.1 ^c^	−4.1 ± 0.0 ^d^	−6.2 ±0.0 ^g^	−3.9 ± 0.0 ^d^	21.8 ± 0.2 ^a^	−5.1 ± 0.0 ^e^
b*	30.9 ± 0.7 ^g^	67.8 ± 0.6 ^a^	54.2 ± 0.6 ^e^	61.4 ± 0.1 ^b^	53.9 ± 0.1 ^d^	53.1 ±0.3 ^de^	53.5 ± 0.2 ^de^	58.2 ± 0.8 ^c^	33.1 ± 0.0 ^f^

Values within the same row that carry distinct superscript letters (a–i) indicate statistically significant differences at *p* < 0.05.

**Table 7:** Texture profile analysis parameters: hardness, adhesiveness, springiness, and cohesiveness, rheological parameters including storage modulus (G′), loss modulus (G″) at 20 °C and 90 °C, maximum loss tangent (Tan δ_max_), and thermal behaviour, i.e., DSC Peak T (°C) of block-style products.

	Cheddar	Processed	Plant 1	Plant 2	Plant 3	Plant 4	Plant 5
Hardness (N)	179.2 ± 2.4 ^a^	80.8 ± 1.6 ^f^	105.9 ± 0.6 ^e^	145.3 ± 2.3 ^b^	131.5 ± 0.6 ^c^	65.7 ± 0.7 ^g^	124.5 ± 2.2 ^d^
Adhesiveness (N.s)	11.1 ± 0.8 ^b^	16.1 ± 4.1 ^a^	0.8 ± 0.3 ^g^	2.1 ± 0.7 ^e^	4.5 ± 0.4 ^d^	1.3 ± 0.1 ^f^	5.1 ± 0.8 ^c^
Springiness (-)	0.2 ± 0.0 ^c^	0.1 ± 0.0 ^d^	0.4 ± 0.1 ^b^	0.5 ± 0.2 ^a^	0.4 ± 0.2 ^b^	0.2 ± 0.0 ^c^	0.2 ± 0.1 ^c^
Cohesiveness (-)	0.1 ± 0.0 ^a^	0.1 ± 0.0 ^a^	0.1 ± 0.0 ^a^	0.1 ± 0.0 ^a^	0.1 ± 0.0 ^a^	0.1 ± 0.0 ^a^	0.1 ± 0.0 ^a^
G′ (20 °C)	56.9 ± 5.6 ^c^	15.1 ± 0.9 ^g^	34.7 ± 4.3 ^e^	23.4 ± 2.8 ^f^	47.3 ± 0.2 ^d^	86.1 ± 16.5 ^a^	70.5 ± 12.9 ^b^
G″ (20 °C)	15.1 ± 0.8 ^a^	3.3 ± 0.5 ^c^	4.7 ± 0.8 ^c^	1.9 ± 0.5 ^c^	4.4 ± 0.4 ^c^	8.8 ± 2.3 ^b^	9.5 ± 2.2 ^b^
G′ (90 °C)	0.1 ± 0.0 ^d^	2.7 ± 0.7 ^a^	0.2 ± 0.0 ^d^	1.2 ± 0.1 ^bc^	0.7 ± 0.1 ^cd^	1.9 ± 0.2 ^b^	1.4 ± 0.3 ^bc^
G″ (90 °C)	0.1 ± 0.0 ^d^	0.8 ± 0.2 ^a^	0.0 ± 0.0 ^e^	0.3 ± 0.0 ^b^	0.2 ± 0.0 ^c^	0.3 ± 0.0 ^b^	0.3 ± 0.1 ^c^
Tan δ_max_	1.9 ± 0.0 ^a^	0.4 ± 0.0 ^b^	0.2 ± 0.0 ^d^	0.3 ± 0.0 ^c^	0.2 ± 0.0 ^cd^	0.1 ± 0.0 ^e^	0.2 ± 0.0 ^de^
DSC Peak 1 T (°C)	13.8 ± 0.0 ^b^	12.0 ± 0.7 ^c^	21.6 ± 0.5 ^a^	21.1 ± 0.3 ^a^	20.8 ± 0.1 ^a^	21.4 ± 0.9 ^a^	21.2 ± 0.5 ^a^
DSC Peak 2 T (°C)	30.1 ± 0.8 ^a^	29.5 ± 0.6 ^a^	-	-	-	-	-

Values within the same row that carry distinct superscript letters (a–g) indicate statistically significant differences at *p* < 0.05.

**Table 8:** Uniaxial compression testing i.e., hardness, along with rheological parameters, including storage modulus (G′), loss modulus (G″) at 20 °C and 90 °C, maximum loss tangent (Tan δ_max_), and thermal behaviour, i.e., DSC Peak T (°C) of slice-style products.

	Cheddar	Processed	Plant 1	Plant 2	Plant 3	Plant 4	Plant 5	Plant 6	Plant 7
Hardness (N)	3.4 ± 0.0 ^ef^	0.6 ± 0.0 ^h^	2.8 ± 0.0 ^g^	3.6 ± 0.0 ^d^	3.3 ± 0.0 ^f^	4.7 ± 0.0 ^b^	3.5 ± 0.0 ^e^	6.6 ± 0.1 ^a^	3.8 ± 0.0 ^c^
G′ (20 °C)	119.4 ± 16.7 ^b^	11.7 ± 2.4 ^f^	57.5 ± 7.1 ^de^	94.3 ± 21.2 ^bcd^	49.6 ± 5.1 ^ef^	72.4 ± 6.7 ^cde^	106.8 ± 21.5 ^bc^	290.4 ± 14.9 ^a^	85.9 ± 7.8 ^bcde^
G″ (20 °C)	32.3 ± 4.3 ^b^	4.3 ± 0.2 ^de^	4.5 ± 1.6 ^cd^	13.9 ± 5.8 ^bc^	2.8 ± 0.3 ^e^	9.7 ± 1.6 ^bcd^	15.6 ± 4.7 ^c^	45.9 ± 5.7 ^a^	11.9 ± 0.3 ^bcd^
G′ (90 °C)	0.0 ± 0.0 ^d^	0.7 ± 0.1 ^cd^	1.9 ± 0.4 ^a^	1.3 ± 0.4 ^abc^	1.5 ± 0.1 ^ab^	1.5 ± 0.2 ^ab^	1.2 ± 0.4 ^bc^	0.4 ± 0.0 ^d^	1.4 ± 0.2 ^ab^
G″ (90 °C)	0.1 ± 0.0 ^b^	0.3 ± 0.0 ^ab^	0.5 ± 0.1 ^a^	0.3 ± 0.1 ^ab^	0.3 ± 0.1 ^ab^	0.4 ± 0.1 ^a^	0.2 ± 0.1 ^ab^	0.1 ± 0.0 ^b^	0.2 ± 0.1 ^ab^
Tan δ_max_	2.5 ± 0.4 ^a^	0.6 ± 0.0 ^b^	0.2 ± 0.0 ^c^	0.2 ± 0.3 ^c^	0.2 ± 0.0 ^c^	0.2 ± 0.0 ^c^	0.2 ± 0.0 ^c^	0.2 ± 0.0 ^c^	0.2 ± 0.0 ^c^
DSC Peak 1 T (°C)	13.3 ± 0.2 ^d^	31.4 ± 0.3 ^a^	20.2 ± 0.2 ^c^	21.0 ± 0.7 ^c^	20.5 ± 0.8 ^c^	20.8 ± 0.9 ^c^	21.3 ± 0.9 ^c^	29.5 ± 0.9 ^b^	21.4 ± 0.8 ^c^
DSC Peak 2 T (°C)	29.2 ± 0.3 ^a^	-	-	-	-	-	-	-	-

Values followed by different superscript letters (a–h) in the same row are significantly different (*p* < 0.05).

**Table 9:** Beta coefficient values for the seonsory data (hedonic and descriptive) for block-style dairy and plant-based cheese analogues.

	Liking of Appearance	Liking of Aroma	Liking of Flavour	Liking of Texture	Overall Acceptability	Colour	Cheddar Aroma	Firmness in Mouth	Pasty Texture	Crumbly Texture	Sweet Taste	Salt Taste	Sour Taste	Bitter Taste	Cream Flavour	Cheddar Flavour	Dairy Sweet Flavour	Dairy Fat Flavour	Fruity (estery) Flavour	Off-Flavour	Astringent Aftertaste
Cheddar	1.83	1.49	2.21	1.83	2.28	2.63	2.62	0.29	1.48	−1.42	0.28	0.22	−0.47	−0.31	0.85	2.43	0.67	1.25	−0.27	−1.22	−0.65
Processed	0.13	0.09	0.15	0.08	0.14	0.50	0.20	−0.25	0.29	−0.21	−0.05	−0.01	0.00	−0.03	0.06	0.18	0.03	0.07	−0.08	−0.07	0.00
Plant 1	−0.27	−0.22	−0.33	−0.27	−0.34	−0.39	−0.39	−0.04	−0.22	0.21	−0.04	−0.03	0.07	0.05	−0.13	−0.36	−0.10	−0.19	0.04	0.18	0.10
Plant 2	−0.23	−0.19	−0.28	−0.23	−0.29	−0.34	−0.33	−0.04	−0.19	0.18	−0.04	−0.03	0.06	0.04	−0.11	−0.31	−0.09	−0.16	0.03	0.16	0.08
Plant 3	−0.20	−0.16	−0.24	−0.20	−0.25	−0.29	−0.29	−0.03	−0.16	0.16	−0.03	−0.02	0.05	0.03	−0.09	−0.27	−0.07	−0.14	0.03	0.14	0.07
Plant 4	−1.53	−1.24	−1.84	−1.53	−1.91	−2.20	−2.19	−0.24	−1.24	1.18	−0.24	−0.18	0.40	0.26	−0.71	−2.04	−0.56	−1.05	0.22	1.02	0.55
Plant 5	−0.98	−0.79	−1.17	−0.97	−1.21	−1.40	−1.40	−0.15	−0.79	0.75	−0.15	−0.12	0.25	0.17	−0.45	−1.30	−0.36	−0.67	0.14	0.65	0.35

**Table 10:** *p*-Values of beta coefficients (figures shaded in green and red represent positive and negative significant correlations [+/− (*p* < 0.05)] respectively, for the seonsory data (hedonic and descriptive) for block-style products.

	Liking of Appearance	Liking of Aroma	Liking of Flavour	Liking of Texture	Overall Acceptability	Colour	Cheddar Aroma	Firmness in Mouth	Pasty Texture	Crumbly Texture	Sweet Taste	Salt Taste	Sour Taste	Bitter Taste	Cream Flavour	Cheddar Flavour	Dairy Sweet Flavour	Dairy Fat Flavour	Fruity (estery) Flavour	Off-Flavour	Astringent Aftertaste
Cheddar	0.00	0.00	0.00	0.00	0.00	0.25	0.00	0.00	0.20	0.75	0.61	0.08	0.11	0.65	0.02	0.00	0.14	0.00	0.43	0.00	0.23
Processed	0.12	0.33	0.01	0.38	0.06	0.00	0.01	0.00	0.00	0.00	0.72	0.69	0.06	0.10	0.30	0.00	0.68	0.24	0.07	0.17	0.51
Plant 1	0.55	0.64	0.57	0.73	0.59	0.07	0.49	0.40	0.33	0.68	0.56	0.94	0.78	0.90	0.82	0.51	0.96	0.84	0.74	0.72	0.79
Plant 2	0.62	0.99	0.00	0.00	0.00	0.00	0.53	0.02	0.02	0.38	0.97	0.37	0.84	0.42	0.10	0.19	0.87	0.46	0.96	0.03	0.26
Plant 3	0.80	0.56	0.20	0.24	0.12	0.36	0.49	0.13	0.37	0.50	0.10	0.36	0.12	0.29	0.92	0.64	0.68	0.56	0.69	0.58	0.32
Plant 4	0.02	0.06	0.07	0.19	0.08	0.00	0.00	0.00	0.71	0.01	0.00	0.61	0.00	0.11	0.36	0.00	0.06	0.00	0.38	0.53	0.05
Plant 5	0.08	0.09	0.01	0.02	0.01	0.00	0.00	0.15	0.02	0.34	0.33	0.65	0.05	0.28	0.61	0.01	0.64	0.35	0.92	0.35	0.38

**Table 11:** Beta coefficient values for the seonsory data (hedonic and descriptive) for slice-style dairy and plant-based cheese analogues.

	Liking of Appearance	Liking of Aroma	Liking of Flavour	Liking of Texture	Overall Acceptability	Colour	Cheddar Aroma	Firmness in Mouth	Pasty Texture	Crumbly Texture	Sweet Taste	Salt Taste	Sour Taste	Bitter Taste	Cream Flavour	Cheddar Flavour	Dairy Sweet Flavour	Dairy Fat Flavour	Fruity (estery) Flavour	Off-Flavour	Astringent Aftertaste
Cheddar	1.98	2.44	2.95	3.12	3.19	−3.35	2.04	0.77	0.31	−0.35	0.29	0.21	0.97	0.43	0.73	2.09	0.80	0.75	0.46	−1.89	−0.24
Processed	−0.49	−0.60	−0.72	−0.76	−0.78	0.82	−0.50	−0.19	−0.07	0.09	−0.07	−0.05	−0.24	−0.11	−0.18	−0.51	−0.20	−0.18	−0.11	0.46	0.06
Plant 1	0.33	0.41	0.49	0.52	0.53	−0.56	0.34	0.13	0.05	−0.06	0.05	0.04	0.16	0.07	0.12	0.35	0.13	0.13	0.08	−0.32	−0.04
Plant 2	−0.69	−0.84	−1.02	−1.08	−1.11	1.16	−0.71	−0.27	−0.11	0.12	−0.10	−0.07	−0.33	−0.15	−0.25	−0.72	−0.28	−0.26	−0.16	0.66	0.08
Plant 3	0.33	0.41	0.50	0.52	0.54	−0.56	0.34	0.13	0.05	−0.06	0.05	0.04	0.16	0.07	0.12	0.35	0.14	0.13	0.08	−0.32	−0.04
Plant 4	−0.19	−0.24	−0.28	−0.30	−0.31	0.32	−0.20	−0.07	−0.03	0.03	−0.03	−0.02	−0.09	−0.04	−0.07	−0.20	−0.08	−0.07	−0.04	0.18	0.02
Plant 5	0.32	0.40	0.48	0.51	0.52	−0.54	0.33	0.13	0.05	−0.06	0.05	0.03	0.16	0.07	0.12	0.34	0.13	0.12	0.07	−0.31	−0.04
Plant 6	−1.10	−1.36	−1.64	−1.73	−1.77	1.86	−1.13	−0.43	−0.17	0.20	−0.16	−0.12	−0.54	−0.24	−0.40	−1.16	−0.45	−0.42	−0.25	1.05	0.13
Plant 7	−0.47	−0.58	−0.70	−0.74	−0.75	0.79	−0.48	−0.18	−0.07	0.08	−0.07	−0.05	−0.23	−0.10	−0.17	−0.49	−0.19	−0.18	−0.11	0.45	0.06

**Table 12:** *p*-Values of beta coefficients (figures shaded in green and red represented positive and negative significant correlations [+/− (*p* < 0.05)], respectively, for the seonsory data (hedonic and descriptive) for slice-style products.

	Liking of Appearance	Liking of Aroma	Liking of Flavour	Liking of Texture	Overall Acceptability	Colour	Cheddar Aroma	Firmness in Mouth	Pasty Texture	Crumbly Texture	Sweet Taste	Salt Taste	Sour Taste	Bitter Taste	Cream Flavour	Cheddar Flavour	Dairy Sweet Flavour	Dairy Fat Flavour	Fruity (estery) Flavour	Off-Flavour	Astringent Aftertaste
Cheddar	0.00	0.00	0.00	0.00	0.00	0.00	0.00	0.08	0.09	0.93	0.62	0.21	0.00	0.19	0.03	0.00	0.06	0.01	0.25	0.00	0.87
Processed	0.01	0.53	0.73	0.11	0.99	0.00	0.24	0.00	0.00	0.00	0.84	0.50	0.82	0.00	0.33	0.36	0.33	0.06	0.51	0.34	0.11
Plant 1	0.40	0.21	0.21	0.71	0.27	0.00	0.83	0.82	0.43	0.14	0.69	0.63	0.20	0.25	0.53	0.26	0.37	0.88	0.33	0.08	0.24
Plant 2	0.07	0.82	0.04	0.07	0.07	0.00	0.72	0.10	0.04	0.10	0.91	0.48	0.03	0.10	0.35	0.09	0.55	0.41	0.09	0.50	0.58
Plant 3	0.07	0.61	0.46	0.43	0.50	0.00	0.81	0.21	0.31	0.95	0.65	0.78	0.90	0.81	0.83	0.93	0.70	0.76	0.86	0.69	0.92
Plant 4	0.18	0.02	0.05	0.34	0.04	0.00	0.10	0.04	0.04	0.05	0.62	0.63	0.78	0.09	0.59	0.56	0.08	0.14	0.32	0.02	0.10
Plant 5	0.10	0.49	0.30	0.46	0.40	0.00	1.00	0.61	0.54	0.59	0.67	0.62	0.70	0.92	0.73	0.81	0.50	0.83	0.98	0.52	0.68
Plant 6	0.01	0.00	0.00	0.01	0.00	0.00	0.71	0.47	0.47	0.05	0.52	0.53	0.44	0.60	0.90	0.78	0.13	0.87	0.98	0.05	0.33
Plant 7	0.47	0.01	0.01	0.03	0.00	0.00	0.00	0.18	0.02	0.24	0.70	0.24	0.53	0.20	0.19	0.02	0.14	0.05	0.37	0.03	0.23

## Data Availability

The original contributions presented in this study are included in the article/Supplementary Materials. Further inquiries can be directed to the corresponding author.

## Supplementary Materials

The following supporting information can be downloaded at: https://www.mdpi.com/article/10.3390/foods14152701/s1. Figure S1. Differential scanning calorimetry thermograms of heating ramp from −40 to 90 °C for block style cheddar (a), processed (b), Plant 1 (c), Plant 2 (d), Plant 3 (e), Plant 4 (f), and Plant 5 (g) products. Figure S2. Differential scanning calorimetry thermograms of heating ramp from −40 to 90 °C for slice-style cheddar (a), processed (b), Plant 1 (c), Plant 2 (d), Plant 3 (e), Plant 4 (f), Plant 5 (g), Plant 6 (h) and Plant 7 (i) products.
