# Supplementary material for: Comparative Analysis of Composition, Texture, and Sensory Attributes of Commercial Forms of Plant-Based Cheese Analogue Products Available on the Irish Market
Source: Foods. 2025 Jul 31;14(15):2701. doi: 10.3390/foods14152701 (PMC12346050; doi:10.3390/foods14152701)
Supplement: Supplementary file 1 [file foods-14-02701-s001.zip › foods-3753193-supplementary.pdf]

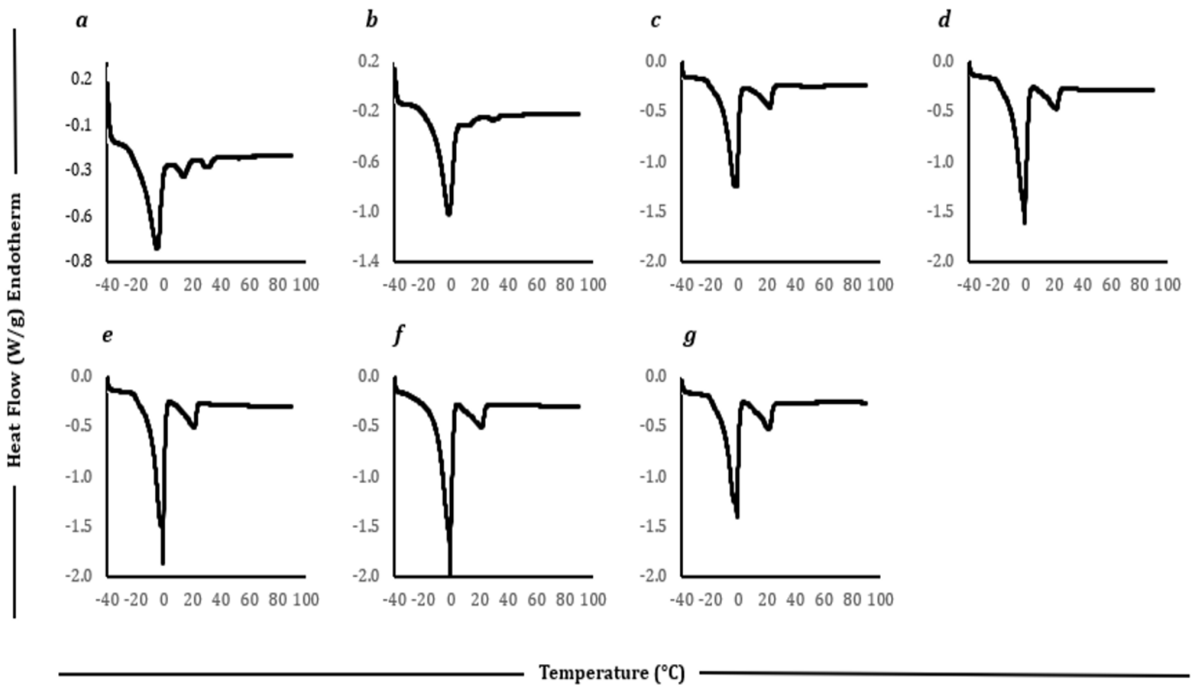

Figure S1. Differential scanning calorimetry thermograms of heating ramp from −40 to 90°C for block style cheddar (a), processed (b), plant 1 (c), plant 2 (d), plant 3 (e), plant 4 (f) and plant 5 (g) products.

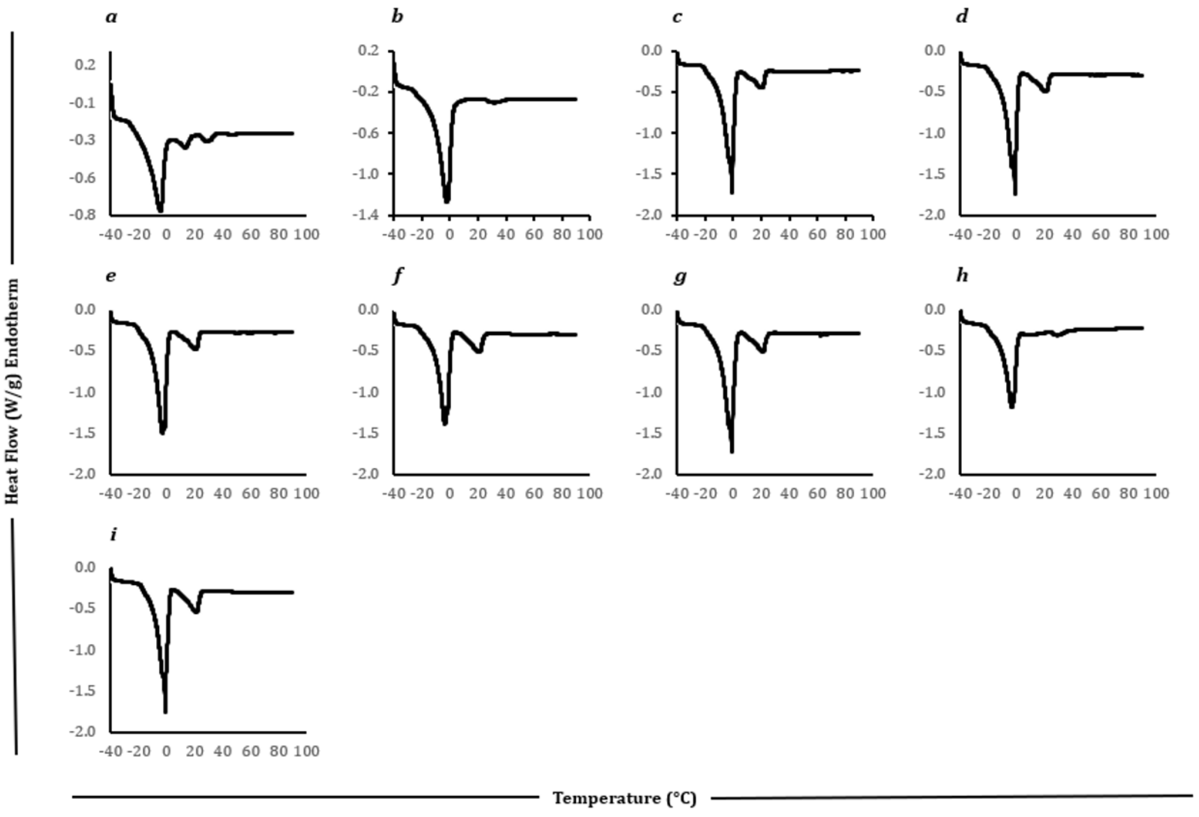

Figure S2. Differential scanning calorimetry thermograms of heating ramp from −40 to 90°C for Slice style cheddar (a), processed (b), plant 1 (c), plant 2 (d), plant 3 (e), plant 4 (f), plant 5 (g), plant 6 (h) and plant 7 (i) products.
